# Proteome of Human Stem Cells from Periodontal Ligament and Dental Pulp

**DOI:** 10.1371/journal.pone.0071101

**Published:** 2013-08-05

**Authors:** Enrica Eleuterio, Oriana Trubiani, Marilisa Sulpizio, Fabrizio Di Giuseppe, Laura Pierdomenico, Marco Marchisio, Raffaella Giancola, Gianluigi Giammaria, Sebastiano Miscia, Sergio Caputi, Carmine Di Ilio, Stefania Angelucci

**Affiliations:** 1 Department of Experimental and Clinical Sciences, G. d'Annunzio University, Chieti-Pescara, Italy; 2 Department of Medical Oral and Biotechnological Sciences, G. d'Annunzio University, Chieti-Pescara, Italy; 3 Department of Medicine and Aging Science, G. d'Annunzio University, Chieti-Pescara, Italy; 4 Center of Aging Science (Ce.S.I.), “Università G. d'Annunzio” Foundation, Chieti, Italy; 5 Stem TeCh Group, Chieti, Italy; 6 Department of Transfusion Medicine, Santo Spirito Hospital, Pescara, Italy; 7 Ravenna Medical Center (GVM Care and Research), Ravenna, Italy; Instituto Butantan, Brazil

## Abstract

**Background:**

Many adult tissues contain a population of stem cells with the ability to regenerate structures similar to the microenvironments from which they are derived in vivo and represent a promising therapy for the regeneration of complex tissues in the clinical disorder. Human adult stem cells (SCs) including bone marrow stem cells (BMSCs), dental pulp stem cells (DPSCs) and periodontal ligament stem cells (PDLSCs) have been characterized for their high proliferative potential, expression of characteristic SC-associated markers and for the plasticity to differentiate in different lineage in vitro.

**Methodology/Principal Findings:**

The aim of this study is to define the molecular features of stem cells from oral tissue by comparing the proteomic profiles obtained with 2-DE followed by MALDI-TOF/TOF of ex-vivo cultured human PDLSCs, DPSCs and BMSCs. Our results showed qualitative similarities in the proteome profiles among the SCs examined including some significant quantitative differences. To enrich the knowledge of oral SCs proteome we performed an analysis in narrow range pH 4–7 and 6–9, and we found that DPSCs vs PDLSCs express differentially regulated proteins that are potentially related to growth, regulation and genesis of neuronal cells, suggesting that SCs derived from oral tissue source populations may possess the potential ability of neuronal differentiation which is very consistent with their neural crest origin.

**Conclusion/Significance:**

This study identifies some differentially expressed proteins by using comparative analysis between DPSCs and PDLSCs and BMSCs and suggests that stem cells from oral tissue could have a different cell lineage potency compared to BMSCs.

## Introduction

Human adult stem cells (SCs), identified in the stromal tissue like bone marrow, spleen, and thymus, are postnatal stem cells able to self-renew and differentiate into multiple cell lineages as bone, cartilage, tendon, skeleton muscle, neuron and oral tissue [1].

Though SCs have a great regenerative ability, their application in dental therapy is still problematic [2].

It is well known that tooth development occurs through mutually inductive signaling between oral epithelial and ectomesenchymal cells originating from migrating neural crest cells, a multipotent cell population derived from the lateral ridges of the neural plate during craniofacial development [3]. Since neural crest cells, contributing to craniofacial bone formation, play a strategic role in tooth organ development, they are considered as a fourth germ layer. Among neural crest cells there are cells with stemness features and multipotency [4].

To date 5 different human dental stem cells have been described in literature: dental pulp stem cells (DPSCs) [5], [6], stem cells from exfoliated deciduous teeth (SHED) [7], periodontal ligament stem cells (PDLSCs) [8], [9], stem cells from apical papilla (SCAP) [10], and dental follicle stem cells (DFSCs) [11]. These cells are intimately associated with dental tissues and easily accessible.

Recently Kim SH et al. [12] and Menicanin et al. [13] compared the gene expression profiles in mesenchymal stem cells derived from different dental tissues and bone marrow to characterize dental stem cell and to provide a dataset of molecules differentially expressed between SCs populations [12] or transcription factors strongly upregulated in all stem cell population examined critical in cell growth and survival [13].

A more accurate and complete pattern of differential gene expression between SCs populations may be derived from proteomic investigations.

Proteomics provides a powerful method to characterize the entire protein profile of stem cell phenotype from different niches. This technology is helpful in understanding the mechanisms that control their self-renewal, differentiation potential and ability to regenerate the unique microenvironments from which they are derived.

In a previous study, Mrozik et al. [14] characterized SCs from ovine periodontal ligament, dental pulp and bone marrow derived from an individual donor and identified differentially expressed proteins to give a molecular description of proteins, crucial for self-renewal and differentiation potential. 58 proteins were differentially expressed in at least two populations of SCs, of which some of them are implicated in neuronal structure and functions [14].

In this work, we performed a typical comparative proteome analysis (2DE approach combined with MALDI–TOF/TOF MS experiments) between human DPSCs, PDLSCs, and BMSCs from different donors to find molecular markers responsible for the regeneration of dental and non-dental structures in stem cell-based tissue engineering protocols.

## Results

### Morphological analyses

**Figure 1:**
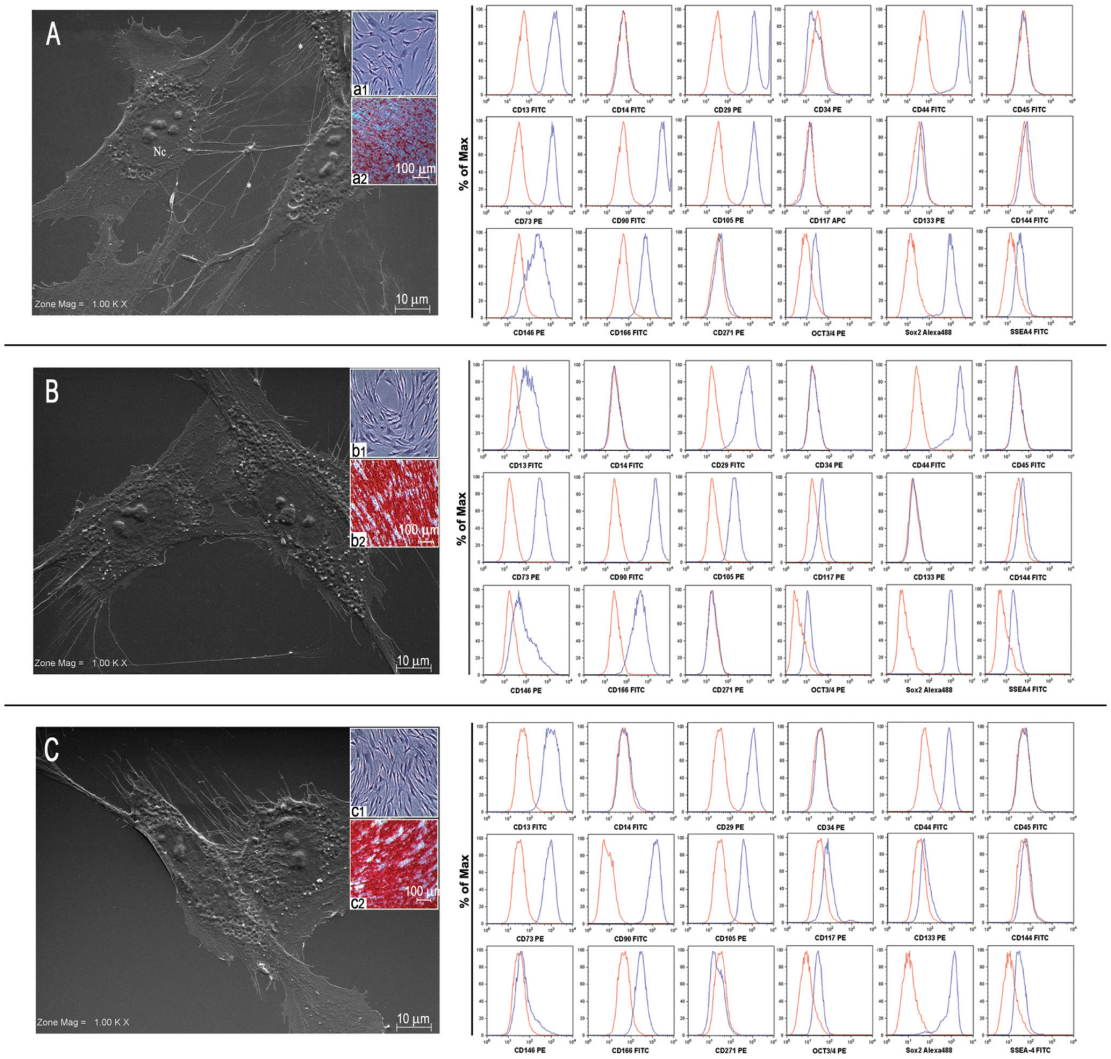
Photomicrographs of primary cultures of BMSCs (A) DPSCs (B) and PDLSCs (C) at passage 2 analyzed at morphological and cytofluorimetric levels. At Scanning (section A, B, and C) and Light microscopy (a1,b1,c1) the culture displays the comparable morphological features consisting of adherent cells having a morphological homogeneous fibroblast-like appearance with a stellate shape and long cytoplasmic processes and numerous filopodia. Nuclei contain one or more nucleoli (Nc). At 28 days of culture in the osteogenic medium BMSCs, DPSCs and PDLSCs (a2,b2,c2) differentiate versus osteoblasts as evidenced by calcium deposition with Alizarin Red staining. Original magnification: 1.000× (scanning microscopy), 10× (light microscopy). The histograms show the cytofluorimetric analysis of the cell culture BMSCs, DPSCs and PDLSCs, surface and intracellular antigens expression profile: CD13, CD14, CD29, CD34, CD44, CD45, CD73, CD90, CD105, CD117 CD133, CD144, CD146, CD166, CD271, OCT3/4, Sox2 and SSEA4. Blue histograms represent cells stained with the expression markers; red histograms show the respective IgG isotype control. These data are representative of four independent biological samples.

In this study we compared BMSCs, DPSCs and PDLSCs at passage 2, when the highest proliferative rate occurs. Under light microscopy, the primary cultures of SCs consisting of colonies of adherent cells showed a morphologically homogeneous fibroblast-like shape. As usual, the cells adhered to each other forming colonies, the nuclei were round or oval-shaped with abundant euchromatin, indicative of an active gene transcription (Fig. 1, insert a1, b1, c1).

The fine structure of BMSCs (Fig. 1, section A), DPSCs (Fig. 1, section B) and PDLSCs (Fig. 1, section C) was analyzed by scanning electron microscopy. At high magnification, primary cultures had a morphologically homogeneous fibroblast-like appearance with a stellate shape and long cytoplasmic processes. The cells showed many filopodia and a secretory apparatus was evident.

### Flow cytometry analysis

The expression of stemness surface molecules (CD13^+^, CD29^+^, CD44^+^, CD105^+^, CD73^+^, CD90^+^, CD146^+^, CD166^+^), and of pluripotency associated markers (OCT3/4^+^, SSEA4^+^, SOX2^+^) was evident in human BMSCs (Fig. 1 histograms A), DPSCs (Fig. 1 histograms B) and PDLSCs (Fig. 1 histograms C). The cells were negative for the following markers: CD117, CD133, CD144, CD271, CD14, CD34, CD45. Table S1 indicate statistical flow cytometry analysis of four different biological samples.

### Cell Growth

**Figure 2:**
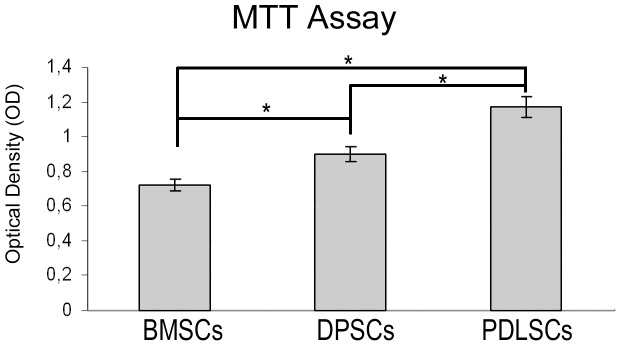
Proliferation rate of the primary culture of BMSCs, DPSCs and PDLSCs plated at passage 2 and after 3 days of culture. The MTT analysis histogram shows that the proliferation in PDLSCs and DPSCs was higher compared to BMSCs. The comparative analysis between the stem cells population demonstrated a difference in terms of cell proliferation to the advantage of stem cells from periodontal ligament. The data represent the mean of four separate experiments carried out in triplicate. Values are reported as mean ±SD (ANOVA test, p≤0.05).

The proliferation rate and viability of the BMSCs, DPSCs and PDLSCs was studied performing a MTT assay at passage 2 after 3 days of culture. The analysis of the obtained data showed a significant cell growth in PDLSCs and DPSCs compared to BMSCs (Fig. 2).

### Osteogenic differentiation

To evaluate the intrinsic capacity of self-renewal and the ability to regenerate tissues of the mesenchymal lineage, an osteogenic differentiation process was induced in primary culture. After 28 days of induction, a mineralized matrix highlighted by alizarin red staining was detected [15]. When we compared the expression intensity of this osteogenic marker, we found that the bone formation was higher in PDLSCs and DPSCs than in bone marrow cells (Fig. 1, insert a2, b2, c2).

### BMSCs, DPSCs and PDLSCs proteome in broad pH range

**Figure 3:**
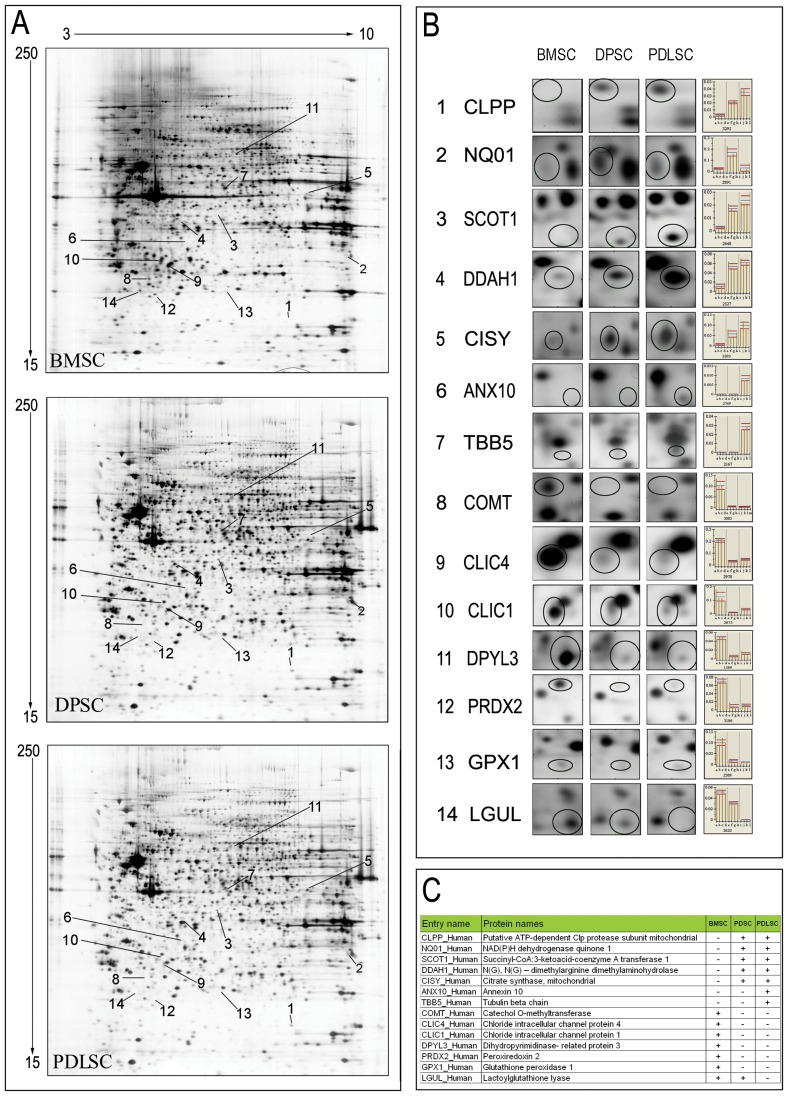
Comparative proteome analysis of BMSCs and oral tissue derived SCs (PDLSCs and DPSCs). Panel A displays the representative 2D pattern of each stem cell population analyzed. Differences are indicated with numbers. Panel B shows magnification of differentially expressed spot (1–14) and relative % of volume histogram. Panel C summarizes proteins differentially expressed in all cell populations examined.

**Figure 4:**
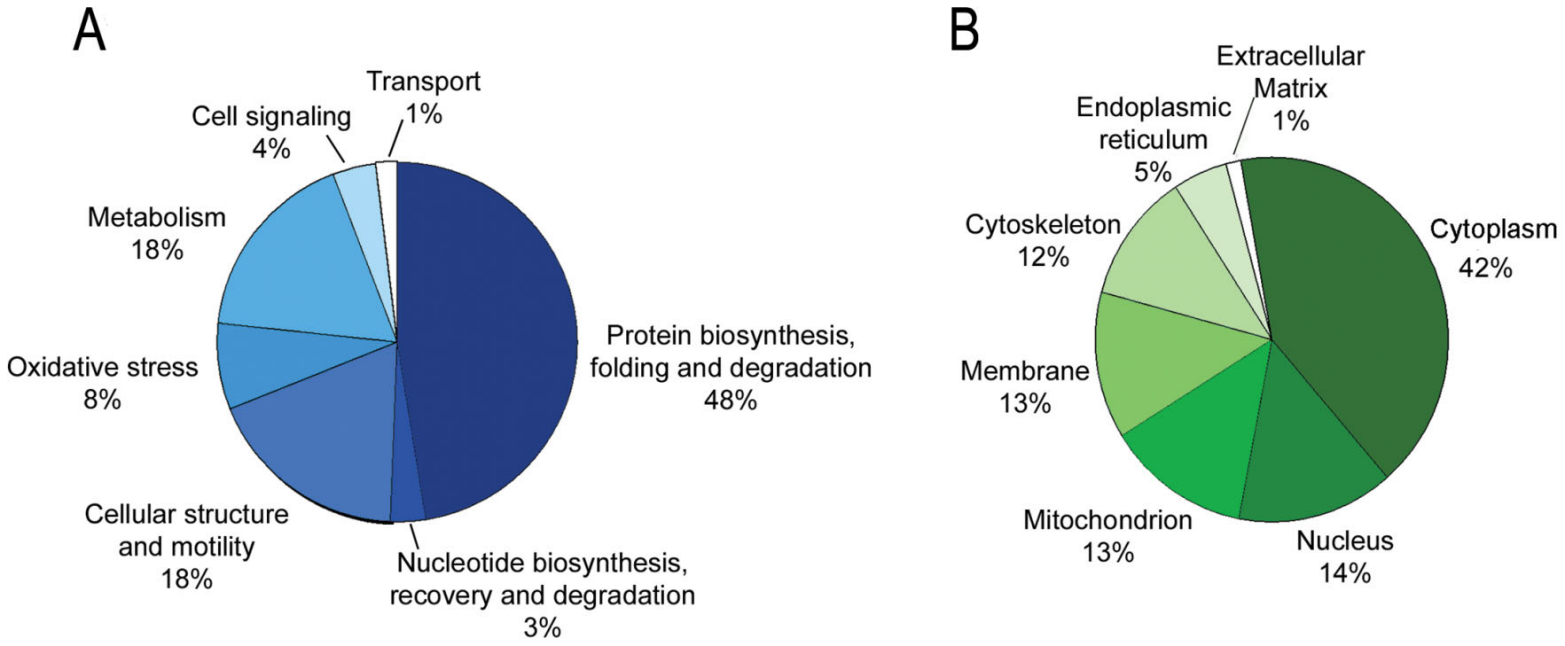
Functional and cellular compartment distribution of oral tissue SCs proteins. The assigned proteins were analyzed using Gene Ontology (GO) annotation and grouped in seven categories based on their functions: 1) protein biosynthesis folding and degradation, 2) cellular structure and motility, 3) metabolism, 4) oxidative stress, 5) cell signaling, 6) nucleotide biosynthesis recovery and degradation, 7) transport. (section A) and in terms of cellular localization (section B).

Comparative proteome analysis of four stem cell populations BMSCs, DPSCs and PDLSCs in the broad pH range (3–10), allowed to detect 2178±376, 2078±357 and 2131±290 spots respectively. SCs show qualitative similarities in the protein profiles. The percentage of matching between gel of the same population was about 81% for BMSCs, 86% for DPSCs and 86% for PDLSCs. The percentage of matching between different populations was about 80% for DPSCs and PDLSCs, 78% for DPSCs and BMSCs and 77% for PDLSCs and BMSCs. Figure 3 (panel A) displays the 2D pattern representative of each stem cell population analyzed. We selected 140 common protein spots on the basis of the highest volume percentage and constant expression level. Subsequently these spots were all picked from gel and digested for mass spectrometric analysis. 113 unique proteins (indicated in figure S1) were identified and listed in table S2. The assigned proteins were analyzed using Gene Ontology (GO) annotation and grouped in seven categories based on their functions: 1) protein biosynthesis folding and degradation, 2) cellular structure and motility, 3) metabolism, 4) oxidative stress, 5) cell signaling, 6) nucleotide biosynthesis recovery and degradation, 7) transport. (Fig. 4 A).

Overall about 48% of total proteome so defined included molecular species belonging to protein biosynthesis and degradation and cellular structure and motility, suggesting that these cells are characterized by a high plasticity and proliferative property. This result is consistent with data literature for stem cell [16]. It is worth noting that overall about 56% of the proteins are distributed in cytosolic and cytoskeletal compartments, suggesting a high stability in cytoarchitecture and structural integrity (Fig. 4 B).

**Figure 5:**
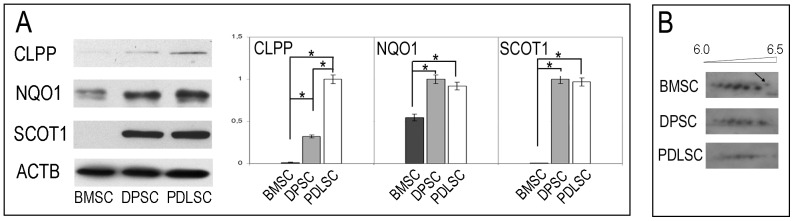
Western Blot analysis of some representative proteins that are differentially expressed between SC populations. A) 1D western blot data for CLPP, NQ01, SCOT1 and ACTB as loading control. Western blot confirms results obtained by 2D analysis. B) 2D western blot data for DPYL3. BMSCs show a higher level of DPYL3 than oral tissue SCs. The new and more basic DPYL3 isoform is indicated by the arrow.

We also found four proteins exclusively expressed in PDLSCs and DPSCs i.e. Putative ATP-dependent Clp protease proteolytic subunit (CLPP), NAD(P)H dehydrogenase (quinone) 1 (NQ01), Succinyl-CoA:3-ketoacid coenzyme A transferase 1 (SCOT1), and an additional new isoform of tubulin (TBB5) (figure 3 panel B and table 1). Our results indicate that N(G),N(G)-dimethylarginine dimethylaminohydrolase 1 (DDAH1) and Citrate synthase (CISY) are up-regulated in PDLSCs and DPSCs compared to BMSCs, as displayed in figure 3. Annexin 10 (ANX10) is present only in PDLSCs. As reported in figure 3 panel B and listed in table 1, PDLSCs and DPSCs do not express Catechol O-methyltransferase 1 (COMT) that is exclusively present in BMSCs. From the PDLSCs and DPSCs proteome analysis, seven proteins i.e. two new isoforms of Chloride intracellular channel protein (CLIC1 and CLIC4), a more basic isoform of Dihydropyrimidinase-related protein 3 (DPYL3) and a Peroxiredoxin-2 (PRDX2) isoform, Glutathione peroxidase 1 (GPX1) and Lactoylglutathione lyase (LGUL), were strongly up regulated in PDLSCs and DPSCs when compared with BMSCs (table 1 and figure 3 panel B). All different expression levels measured for proteins showing up- or down regulation among all cell sample preparations are statistically significant. (Student's t-test with p<0.05). The expression of selected identified proteins were confirmed by 1 e 2D western blot analysis (Fig. 5).

**Table 1:** Differentially espressed proteins in PDLSCs, DPSCs and BMSCs.

pH range	Ertry name	Protein name	Accession Number	Score	% sc	# peptide matched	Exp pI_MW	Theor pI_MW.
**3–10**	CLPP	Putative ATP-dependent Clp protease proteolytic subunit, mitochondrial	Q16740	113	22	7	5.70_24520	8.27_30446
	NQ01	NAD(P)H dehydrogenase [quinone] 1	P15559	154	44	15	9.01_29235	8.91_30905
	SCOT1	Succinyl-CoA:3-ketoacid-coenzyme A transferase 1. mitochondrial	P55809	100	25	9	6.10_34664	7.14_56578
	DDAH1	N(G).N(G)-dimethylarginine dimethylaminohydrolase 1	O94760	82	27	7	5.77_35597	5.53_31444
	ANX10	Annexin A10	Q9UJ72	70	37	7	5.89_35562	5.00_37800
	TBB5	Tubulin beta chain	P07437	159	36	18	6.03_41694	4.78_50095
	COMT	Catechol O-methyltransferase	P21964	111	44	8	5.44_26369	5.26_30474
	CLIC4	Chloride intracellular channel protein 4	Q9Y696	113	71	13	5.59_25450	5.45_28982
	CLIC1	Chloride intracellular channel protein 1	O00299	100	34	7	5.40_30028	5.09_27248
	DPYL3	Dihydropyrimidinase-related protein 3	Q14195	180	44	19	6.27_62223	6.04_62323
	PRDX2	Peroxiredoxin-2	P32119	128	47	9	5.47_22340	5.66_22049
	GPX1	Glutathione peroxidase 1	P07203	136	56	9	6.12_22710	6.15_22360
	LGUL	Lactoylglutathione lyase	Q04760	134	39	10	5.35_20401	5.12_20992
	CISY	Citrate synthase. mitochondrial	O75390	95	22	10	7.78_40085	8.45_51908
**4–7**	CAPG	Macrophage-capping protein	P40121	120	45	15	6.00_39059	5.82_38760
	STMN1	Stathmin	P16949	113	53	12	5.35_17618	5.76_17292
	LEG1	Galectin-1	P09382	70	48	5	5.37_15111	5.34_15048
**6–9**	AK1C1	Aldo-keto reductase family 1 member C1	Q04828	74	30	9	8.06_38859	8.02_37221
	SEP11	Septin-11	Q9NVA2	102	36	12	6.32_50604	6.36_49562
	NECP2	Adaptin ear-binding coat-associated protein 2	Q9NVZ3	77	39	7	8.28_31595	8.49_28435

### DPSCs and PDLSCs proteome in narrow pH ranges (4–7, 6–9)

To enrich knowledge of oral stem cell proteome we carried out an analysis in narrow acidic (4–7) and basic (6–9) pH range.

**Figure 6:**
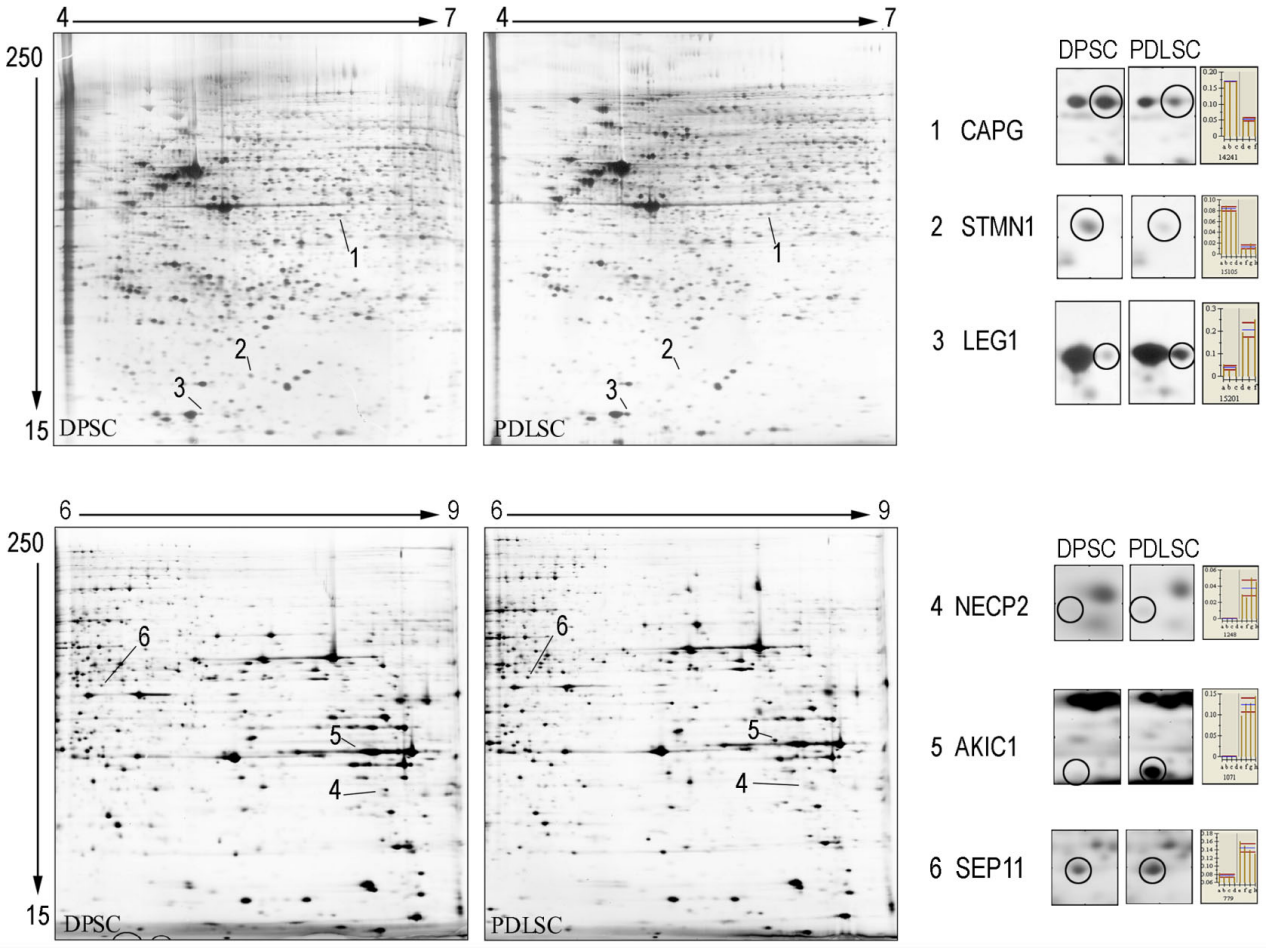
Acidic and basic oral tissue SCs proteome. DPSCs and PDLSCs narrow range (4–7 and 6–9 pH range) 2D maps. Differences are indicated with numbers. Magnifications at the right side show differentially expressed protein spots and relative % volume histograms.

In the 4–7 pH range 1905±101 and 1776±152 protein spots for DPSCs and PDLSCs respectively were resolved, whereas in the 6–9 pH range 1144±187 and 1101±108 protein spots for DPSCs and PDLSCs were resolved. Figure 6 displays the 2D pattern representative of DPSCs and PDLSCs populations analyzed in narrow range (4–7 and 6–9 pH range).

4–7 pH range analysis showed that DPSCs presented a higher expression level of statmin 1 (STMN1) and macrophage-capping protein (CAPG) than galectin 1 (LEG1) which resulted down-regulated.

In 6–9 pH range analysis we found that PDLSCs exclusively express Adaptin ear-binding coat-associated protein 2 (NECP2), and Aldo-keto reductase family 1 member C1 (AK1C1) and septin 11 (SEP11) was up-regulated compared to DPSCs.

## Discussion

Recent studies have suggested that human bone marrow and dental pulp as well as periodontal ligament tissue contain a niche of postnatal stem cells able to differentiate in various cell types as osteoblasts, odontoblasts, cementoblasts, adypocites and neuronal cells [8]–[10], [17]–[20].

Cellular characterization showed that the primary culture from human dental pulp and periodontal ligament are similar to BMSCs for their high clonogenic potential and for expression of embryonic and mesenchymal markers.

Morphological analysis demonstrated that BMSCs, PDLSCs and DPSCs exhibited fibroblast-like morphology after in vitro expansion as previously described [18]. Moreover, oral stem cells versus BMSCs, show a higher proliferative ability from MTT assay results.

GO analysis of representative common protein spots among PDLSCs and DPSCs and BMSCs revealed that the main functional categories are related to protein biosynthesis, folding and degradation and cellular structure and motility. This finding strongly suggests high plasticity, proliferative ability and differentiation potency of these stem cells. In addition, a Heat Shock Protein family (HSPs) expressed at a significant level in both PDLSCs and DPSCs, could play a crucial role in stem cell biology during cellular homeostasis and development process [14], [16].

The high level of common proteins identified in all stem cell analyzed and involved in energetic metabolism belong to the glycolitic pathway suggesting that these stem cells possess a large ability to adapt to anerobic conditions. (Table S2),

All stem cells examined express ubiquitin C-terminal hydrolase, a neuronal deubiquitinating enzyme strongly involved in maintaining neuronal structure function and health [14]. With reference to molecular function and cellular localization, a remarkable finding was the presence of proteins involved in cell structure and motility myosin, tropomyosin, tubulin, vinculin, actin related protein 3, calponin, actin, laminin, cofilin and profilin, that are essential for cytoskeletal organization and morphogenesis implicated in lineage commitment [16], [21]. We also identified two isoforms of vimentin, class-III intermediate filaments found in various non-epithelial (mesenchymal cells and fibroblasts) and common to other SCs (cord blood derived non hematopoietic progenitor cells), and four proteins like tropomyosin, vinculin, actin related protein 3 and calponin 3 that are considered putative markers of differentiation potency versus myocyte and hepatic phenotype [22]–[26].

Annexin 1, 2, and 5, previously selected as markers of SCs, three peroxiredoxin isoforms [1], [2], [6] and Elongation factor Tu, nucleophosmin, a marker of embryonic stemness are interesting proteins expressed in these SCs. [16], [23]–[25], [27].

In table S2 we noted that the major energetic pathway identified is the glycolitic one suggesting that these cells possess a great ability to adapt to anerobic conditions.

Focusing our attention on protein differences based on molecular function and cellular localization we selected and assigned 4 proteins that are not expressed in BMSCs (table 1) i.e. CLPP, NQO1, SCOT1, a new isoform of TBB5 and DDAH1. Taken all together these proteins are implicated in cell cycle regulation and stress response, homing, detoxification and neurogenesis and neuronal function homeostasis [28]–[34].

CLPP belongs to the ClpP family, a mitochondrial serine-proteases which takes part in building the ClpXP (or ClpAP) enzymatic complex. It is considered an important archetypal AAA^+^ proteolytic machine in cells and it is involved in cell cycle regulation and stress response. The process involves degrading multiple cell division proteins, which is a fundamental step for avoiding malignant transformation and necessary for normal cell replication [28].

In addition PDLSCs and DPSCs specifically express NQO1, an antioxidant enzyme with multiple functions (proteasomal degradation and microtubules stabilization), that plays a cytoprotective role [29].

Our findings revealed proteins involved in neurogenesis and neuronal function and homeostasis i.e. SCOT1 and DDAH1 confirming the neuroectodermal origin of oral tissue [30]–[34]. Specifically SCOT1 is a key enzyme for ketone body catabolism, such us acetoacetate and d-3-hydroxybutyrate an important substrate for cerebral metabolism in development and in stress conditions, especially in response to hyperketonemia during the first stages of brain development [30], [31].

DDAH1 regulates the embryonic development of several organ systems including the brain [32] and provides neuronal survival and regeneration after nervous injury [33]. The influence of DDAH1 on control nitric oxyde synthesis by methyl-L-arginines metabolism seems to reduce the ability of stem cells to differentiate in osteoblasts [34].

Chloride intracellular channel proteins (CLICs), as globular and integral membrane proteins are involved in the regulation of the cell cycle [35]. The molecular effects of CLICs on commitment towards osteoblast, keratinocyte, and of fibroblast into myofibroblast has been described [36]–[38]. The down-regulation of CLICs observed in PDLSCs and DPSCs seems to indicate different cell lineage fates of DPSCs and PDLSCs respect to BMSCs. Moreover CLIC1 regulate integrin cell surface expression and is responsible in stem cell homing [39]. Its low expression in DPSC and PDL vs BMSCs seems to remark the different origin of oral stem cells.

Oral stem cells if compared with BMSCs lack a basic isoform of DPYL3. DPYL3 also named Collapsin response mediator protein 4 (CRMP-4) is a specific tissue protein mainly expressed in heart and skeletal muscles that is used as a marker of early neuronal differentiation of hESCs [40] and cultured mouse cerebellar neuron and PC12 [41]. It is important in neurogenesis, by maintaining neuronal structure function, cell migration and cytoskeleton remodelling [40], [41].

A noteworthy result is the identification of ANX10 only in the PDLSCs stem cells. The biological role of ANX10 is not clear since ANX10 deviates from the annexin family by having only one functional Ca2+-binding motif. How this affects the membrane-binding or membrane aggregation properties and thereby the function of ANX10 is unclear so further investigations are needed [42].

Analyzing DPSCs and PDLSCs proteome by using a narrower pH range (4–7 and 6–9) we identified STMN1, CAPG, LEG1, NECP2, AK1C1 and SEP11 that are differentially expressed [43]–[46].

STMN1 is a cytoskeleton-associated protein [43], and is highly expressed in different brain anatomical zones [45]. hESCs-derived neuroectodermal spheres express high level of STMN1 concurrently with neural stem cell markers, Nestin and Poly-Sialated Neural Cell Adhesion Molecule [45]. STMN1 is also involved in neurogenesis process i.e. development, plasticity, degeneration, aging [46] and in post brain ischemic regeneration process [43].

CAPG is a calcium-sensitive protein that modulates cell motility and it is expressed in different cell types i.e. macrophages, neutrophils, fibroblasts and endothelial cells [47]–[49]. CAPG is also involved in multiple differentiation steps such us neuronal and osteogenic process. In fact it was also described as a potential transcriptional repressor in neurons involved in the differentiation of commissural neurons in the spinal cord [48]. CAPG over-expression in human preosteocytic cells versus. pre-osteoblastic cells, suggests its key role in bone differentiation process [47], [49].

SEP11 is expressed in various tissues, including the brain. SEP11 is associated with GABAergic synapses and accumulates in dendritic branching points and at the base of dendritic protrusion of cultured neurons, regulating neuron cytoarchitecture [50].

Another differentially expressed protein in oral stem cells is LEG1 that regulates apoptosis, cellular proliferation and differentiation, binding beta-galactoside and a wide array of complex carbohydrates [51]. LEG1 is expressed in the brain, liver, kidney and pancreas, and at low levels in skeletal muscle and has a functional role in a subset of adult Neural stem cell (NSCs). Sakaguchi et coll. [52] define LEG1 as a potential target of drug design to be used in brain injury and nervous system disorders [52].

Other three basic proteins, NECP2, AK1C1 and SEP11 are differentially expressed in oral tissue derived stem cells.

NECP2 is ubiquitous proteins belonging to NECAP family involved in endocytosis [53]. A very interesting finding is the work of Ritter et al. [53] that for the first time discovered the presence of NECPs in clathrin coated vesicles isolated from rat brain.

AK1C1 also named 20alpha-hydroxysteroid dehydrogenase mRNA was found in liver, prostate, testis, adrenal, brain, uterus and mammary-gland tissues and in human keratinocyte cell and it is involved in steroid pathway [54]. Up to now the molecular function of AK1C1 in SCs biology is still unclear.

In conclusion, comparative analysis between dental pulp and periodontal ligament SCs and bone marrow SCs identifies some differentially expressed proteins and seems to suggest that stem cells from oral tissue could have a different cell lineage potency compared to BMSCs.

Moreover, narrow range pH analysis carried out on oral SCs discovered proteins potentially related to growth, regulation and genesis of neuronal cells suggesting that oral SCs may possess a potential neuronal differentiation ability consistent with their neural crest origin.

SCs may promote neural regeneration by replacing injured neurons via cell differentiation [55], [20]. In an appropriate microenvironment DPSCs present a neural potential, which gives them a neural-like morphology and they significantly over-express most neural markers fundamental for neuroplasticity in cell transplantation [20]. Moreover PDLSCs are able to differentiate into neurogenic lineage [56] and can promote nerve regeneration by secretion of NGF and probably other synergistic factors useful in dental tissue regeneration including nerve repair [55], [56].

In summary SCs from oral tissue represent an easy accessible and autologous niche of stem cells that could provide an advantageous source for regenerative medicine including many tissue systems and nerve repair. Further investigations will be needed to elucidate the mechanisms that control neuronal differentiation of oral SCs.

## Materials and Methods

### Apparatus and reagents

IPG strip gels were run on IPGphor (GE Healthcare, Uppsala, Sweden). Casting and running second-dimensional SDS-PAGE (Dodeca Cell) were purchased from Bio-Rad Laboratories (Hercules, CA, USA). Silver-stained gels were scanned using a Lab Scan (GE Healthcare, Uppsala, Sweden) and image analysis was carried out using Image Master 2D Platinum 6.0 software (GE Healthcare, Uppsala, Sweden). C18ZipTip was purchased from Millipore (Bedford, MA, USA).

Bruker-Daltonics AutoFlex Speed TOF-TOF LIFT Mass Spectrometer (Bruker-Franzen, Germany). Immobiline DryStrip (3–10 NL) 18 cm, pharmalite 3–10, protease inhibitor mix, Drystrip cover fluid, IPG buffer, DeStreak reagent, 2D Clean up Kit and 2D Quant kit reagent were purchased from GE Healthcare (Uppsala, Sweden). Sequencing grade, modified porcine trypsin were obtained from Promega (Madison, WI, USA). All other chemicals were of analytical reagent grade and purchased from Sigma Chemical (St. Louis, MO, USA). All buffers were prepared with Milli-Q water system (Millipore Bedford, MA, USA).

Antibodies used: polyclonal from rabbit vs. CLPP, NQO1, SCOT1, ACTB, (Santa Cruz Biotechnology Inc., Santa Cruz, CA), polyclonal from rabbit DPYL3 (also named CRMP4) were purchased from Abcam, (Cambridge Science Park, UK) and anti-rabbit HRP conjugate (GE Healthcare, Uppsala, Sweden).

### Isolation of Human BMSCs

Four Bone Marrow aspirates were used in this study. The Istituto Superiore di Sanità (ISS) approved the use of bone marrow aspirates in this study (N° 35499-Pre 21-809). Samples were taken for routine diagnostic purposes from the Department of Transfusion Medicine, Santo Spirito Hospital, Pescara, Italy which follows procedures in agreement with ISS guidelines. All donors were recruited according to ISS guidelines and gave their written informed consent for this study authorizing the explant of bone marrow and the use of these cells. All bone marrow samples were de-identified. Bone marrow aspirates were washed with control medium consisting of low glucose Dulbecco's modified Eagle's medium (DMEM-LG, Invitrogen) supplemented with 10% fetal bovine serum (FBS). The procedures for establishing human bone marrow-derived SC cultures followed previously published methods [57]. Density-gradient (Ficoll-Hypaque, GE Healthcare, Milan, Italy) separation was performed to isolate mononuclear cells. Mononuclear cells were resuspended with MSCBM medium (Lonza Verviers Company, Belgium) and seeded at a density of 5×10^3^ cells/cm^2^ to establish primary cultures. The medium was changed twice a week and, as the culture reached around 80% confluence, cells were trypsinized and subsequently subcultured until passage 2.

### Isolation of Human PDLSCs and DPSCs

Four human periodontal ligament biopsies were carried out in human premolar teeth, scheduled to be removed for orthodontic purposes on four healty volunteers aged 20–35 years. All patients provided written consent for clinical research and for the processing of personal data. All periodontal ligament biopsies were de-identified. The peridontal ligament tissue was collected after tooth extraction. Explants were obtained from alveolar crestal and horizontal fibers of the periodontal ligament by scraping the roots of non-carious third molar teeth with a Gracey's curette [58].

Four human premolar teeth, scheduled to be removed for orthodontic purposes, were selected from four healthy patients ranging from 20 to 35 years old. All patients provided written consent for clinical research and for the processing of personal data. All teeth samples were de-identified. Each subject was pre-treated for a week with professional dental hygiene. Extracted teeth were rinsed in phosphate-buffered saline (PBS) containing penicillin and streptomycin. Subsequently, dental pulps were exposed using a cylindrical diamond rotary cutting instrument (Intensiv 314, Ø ISO 014, L.8.0 mm; Intensiv, Grancia, Switzerland) mounted on a high-speed handpiece (Bora L; Bien-Air, Bienne, Switzerland) with water-spray cooling. Pulps were then extracted with a sterile dentinal excavator and cut into small pieces and cultured in MSCM medium (Lonza Verviers Company, Belgium), according to established methods [59]. The PDLSCs and DPSCs were obtained and cultured in MSCM medium (Lonza Verviers Company, Belgium) according to Trubiani et al. [59]. Cells migrated from the explants and on day 7, adherent cells which were 80–90% confluent as determined by phase contrast microscopy, were isolated using 0.1% trypsin solution and plated in tissue culture polystyrene flasks at 5×10^3^ cells/cm2.

### Cell proliferation assay

Human expanded ex vivo BMSCs, DPSCs and PDLSCs were seeded at passage 2 (1×10^3^ cells/well) in triplicate using a 96-well flat-bottom plate and maintained in MSCM medium for 3 days. After the incubation period, 15 µl/well of MTT was added to culture medium and cells were incubated for 3 h at 37°C. The supernatants were read at 650 nm wavelength using an ND-1000 NanoDrop Spectrophotometer (NanoDrop Technologies, Rockland, DE, USA).

The primary cultures of BMSCs, DPSCs and PDLSCs at passage 2 were used for all Scanning Electron Microscopy (SEM) experiments. The samples were prefixed for 4 h at 40°C in 2% glutaraldehyde in 0.05M phosphate buffer (pH 7.4), post-fixed in 1% OsO_4_, dehydrated in increasing ethanol concentrations and then critical point-dried. They were then mounted on aluminum stubs and gold-coated in an Emitech K550 sputter-coater (Emitech Ltd. Ashford, Kent, UK) before imaging by means of a SEM (LEO 435 V, Cambridge, UK).

### Flow cytometry analysis of SCs

BMSCs, DPSCs and PDLSCs at the passage 2 were treated with 0.1% trypsin-EDTA, harvested and suspended in PBS and stained with the following markers: Fluorescein isothiocyanate-conjugated anti-CD13 (CD13 FITC), phycoerythrin-conjugated anti-CD29 (CD29 PE), FITC-conjugated anti-CD44 (CD44 FITC), FITC-conjugated anti-CD45 (CD45 FITC), FITC-conjugated anti-CD105 (CD105 FITC) and FITC-conjugated anti-CD166 (CD166 FITC) were obtained from Ancell (MN, USA); FITC-conjugated anti-CD14 (CD14 FITC) and PE-conjugated anti-CD133 (CD133 PE) were purchased from Miltenyi Biotec (Bergisch Gladbach, Germany); PE-conjugated anti-CD73 (CD73 PE), FITC-conjugated anti-CD90 (CD90 FITC), allophycocyanin-conjugated anti-CD117 (CD117-APC), PE-conjugated anti-CD146 (CD146 PE), PE-conjugated anti-CD271 (CD271-PE), Alexa488-conjugated Sox2 (Sox2 Alexa488), FITC-conjugated anti-SSEA4 (SSEA4 FITC) and PE-conjugated anti-OCT3/4 (OCT3/4 PE) obtained from Becton Dickinson (BD, San Jose, CA); FITC-conjugated anti-CD144 (CD144-FITC) was obtained from Acris Antibodies (Herford, Germany); PE-conjugated anti-CD34 (CD34-PE) was purchased from Beckman Coulter (Fullerton, CA, USA); appropriate secondary FITC-conjugated antibody was obtained from Jackson Immunoresearch Laboratories (West Grove, PA, USA). Washing buffer (PBS, 0.1% sodium azide and 0.5% bovine serum albumine), was used for all washing steps. Samples were stained for surface or intracellular antigens, as previously described [60]. 5×10^5^ cells/sample were incubated with 100 µl of 20 mM ethylenediamintetraacetic acid (EDTA) at 37°C for 10 min. Cells were washed with 3 ml of washing buffer and centrifuged (4°C, 400× g, 8 min). For surface antigens, samples were resuspended in 100 µl washing buffer containing the appropriate amount of surface antibodies, incubated for 30 min at 4°C in the dark, washed (3 ml of washing buffer), centrifuged (4°C, 400× g, 8 min), resuspended with 1 ml 0.5% paraformaldehyde, incubated for 5 min at RT, washed, centrifuged (4°C, 400× g, 8 min) and stored at 4°C in the dark until acquisition. For intracellular antigens, samples were resuspended in 1 ml of FACS Lysing solution (BD), vortexed and incubated at room temperature in the dark for 10 min. Samples were centrifuged (4°C, 400× g, 8 min); 1 ml of Perm 2 (BD) was added to each tube and cells were incubated at RT in the dark for 10 min. Samples were washed and centrifuged (4°C, 400× g, 8 min). Cells were resuspended in 100 µl of washing buffer containing the appropriate amount of intracellular antibodies and incubated for 30 min at 4°C in the dark. Cells were centrifuged (4°C, 400× g, 8 min), resuspended with 1 ml 0.5% paraformaldehyde, incubated for 5 min at RT, washed, centrifuged (4°C, 400× g, 8 min) and stored at 4°C in the dark until the acquisition. Cells were analysed on a FACSCalibur flow cytometer (BD), using CellQuest™ software (BD).

Quality control included regular check-ups with Rainbow Calibration Particles (BD Biosciences). Debris was excluded from the analysis by gating on morphological parameters; 20,000 non-debris events in the morphological gate were recorded for each sample. To assess non-specific fluorescence we used isotype controls. All antibodies were titrated under assay conditions and optimal photomultiplier voltages (PMT) were established for each channel [61]. Data were analyzed using FlowJo™ software (TreeStar, Ashland, OR).

### Analysis of BMSCs, DPSCs and PDLSCs differentiation into osteogenic lineage

For osteogenesis induction, the primary cultures were seeded at 4×10^3^ cells/cm^2^ in MSCBM culture medium and maintained in culture at 37°C, in a humidified atmosphere of 5% CO_2_. After reaching a subconfluence level, cells were incubated with osteogenesis MSCBM induction medium (Lonza Company, Belgium). A fresh medium was added twice a week, and after 28 days of culture, osteogenic differentiation was assessed by alizarin red staining. Cells were fixed with parafomaldheyde 4%, washed three times with PBS (pH 7.4), then stained with 0.5% alizarin red S in H_2_O, pH 4.0, for 1 h at room temperature. After staining, the cultures were washed three times with H_2_O followed by 70% ethanol.

### 2DE analysis

Four samples for each population of BMSCs, DPSCs and PDLSCs were centrifuged at 1200×g and washed in ice-cold PBS three times. Total proteins were extracted from 10^7^ cells with 500 µl of lysis buffer (8 M urea, 4% CHAPS, 40 mM Tris and 2 mM TBP and a mixture of protease inhibitors purchased by GE Healthcare (Uppsala, Sweden). The extraction mixture was sonicated three times for 20 s with 40% amplitude by using U200S sonicator (IKA Labortechnik, Germany) then centrifuged at 14 000×g for 20 min and incubated with endonuclease (150 U) for 20 min at room temperature. Samples were purified with 2D Clean up Kit (GE Healthcare, Uppsala, Sweden) before loading. Protein concentrations were determined using Better Bradford (Pierce), in accordance to the manufacturer's instructions.

Samples were run in triplicate and were separated by using Immobiline DryStrip IPG strip 18 cm 3–10 NL for the first dimension and SDS-PAGE 9–16% for the second dimension according to procedures previously described by Angelucci et al. [21]. IEF was performed on an IPGphor IEF system (GE Healthcare, Uppsala, Sweden) using the following program: two steps of active rehydration of 30 V for 2 h and 60 V for 6 h, respectively; then 1 h at 500 V, 1 h at 1000 V, 8000 V for 30 min by gradient; finally 8000 V for 4 h 30 min by step and hold until total amount of 70000 Vh was obtained.

A total amount of 100 µg (for the analytical gels) and 500 µg (for the preparative gels) of total proteins were loaded on the non linear immobilized pH gradient strips.

Analytical gels were stained as previously described [21]. Staining gels were scanned at 600 dpi with LabScan 5.0 (GE Healthcare, Uppsala, Sweden) and then analyzed with Image Master 2D Platinum 6.0 (GE Healthcare, Uppsala, Sweden). Spots were detected with the following parameters: smooth 3, minimal area 48 and saliency 200. A positional gel calibration was carried out by using a 2D calibration method included in the analysis package that calculates the position of protein spots in terms of their isoelectric point (pI) and Molecular Weight (MW) values. Several spots, Heat shock protein Hsp 90-alpha, Peroxiredoxin1, Proteasome subunit beta type 3 (Mr 84875 pI 4.94, Mr 22324 pI 8.27, Mr 23219 pI 6.14) identified by mass spectrometry analysis were spread throughout the gels and were chosen as marker proteins. For each sample we performed triplicate 2DE-gels. Proteins with quantitative density differences were analyzed for statistical significance using a Student's t test (p<0.05). Proteins that were significantly regulated between the same SCs population were excised for identification.

### Protein digestion and MALDI-TOF/TOF-MS

Following differential analysis, protein spots on preparative 2-DE gels were excised and analyzed by the peptide mass finger printing (PMF) approach with a MALDI-TOF/TOF Mass spectrometer and by mass list probabilistic matching by MOWSE score algorithms on the Swiss-Prot database.

Protein spots were excised from the gel and were washed with 100% ethanol and 100 mM ammonium bicarbonate (NH_4_HCO_3_). They were then incubated for 60 min at 56°C in 100 µl of 50 mM NH_4_HCO_3_ supplemented with 10 mM DTT and then for 30 min in the dark in 100 µl of 50 mM NH_4_HCO_3_ supplemented with 55 mM iodoacetamide at room temperature. Subsequently the gel was reswollen in 50 mM NH4HCO3 containing 12.5 ng trypsin and incubated at 37°C overnight [62]. Peptide extract was applied to a C18ZipTip (Millipore, Bedford, MA, USA), rinsed with a 0.1% TFA and eluted directly on the MALDI target with 0.5 µl of a saturated α-cyano-4-hydroxycinnamic acid (1∶1 = ACN:0.1% TFA) solution. All analyses were carried out in reflex positive ion mode at an accelerating voltage of 20 kV and a reflex voltage of 23 kV.

The instrument was calibrated with external standards such as bradykinin (fragment 1–7) 757.39 m/z, angiotensin II 1046.54 m/z, ACTH (fragment 18–39) 2465.19 m/z, Glu fibrinopeptide B 1571.57 m/z, and renin substrate tetradecapeptide porcine 1760.02 m/z. Internal mass calibration was performed using trypsin autodigestion products (842.50 m/z, 1045.56 m/z, 2211.11 m/z, 2283.19 m/z). The peptide mass fingerprints (PMF) obtained were used to search through the SWISS-PROT and NCBInr databases using the Mascot search engine (http://www.matrixscience.co.uk) with a tolerance of 100 ppm and one missed cleavage site.

### Western blot analysis

An equal protein amount of 20 µg for 1D and 2D from each sample cell were separated by electrophoresis and transferred to nitrocellulosa. For 2DE the samples were separated on 7 cm pH 3–10 no-linear IPG strips (GE Healthcare, Uppsala, Sweden). IEF was performed by a 12-h in-gel rehydration at 50 V, followed by focusing at 5000 V for a total of 20 kVh. The strips were incubated in equilibration buffer (8 M urea, 2% SDS, 30% (v/v) glycerol, 50 mM Tris–HCl, pH 8.8), reduced with 1% (w/v) DTT for 15 min, and alkylated with 2.5% iodoacetamide for 15 min in darkness. The strips were separated in the 2nd dimension by 12% SDS-PAGE and were transferred to nitrocellulose at 50 V for 12 h at 4°C. Western blot analysis was carried out according to standard procedures [63] by using the primary antibodies diluted 1∶1000 and secondary antibodies diluted 1∶3000. Blots were developed with ECL Plus (GE Healthcare) and visualized by autography on Biomax light film Sigma Chemical (St. Louis, MO, USA). In order to avoid changes in time exposure, 2D blots of samples to be compared were exposed in the same cassette and the results were visualized on the same film. Monodimensional bands were quantified by Quantity One Bio-Rad and spots from 2D blot were quantified by Image Master 2D Platinum normalizing for βactin.

## Supporting Information

Figure S1 **Localization on 2D map of high abundant proteins listed in table S2 and indicated by entry name.**
(TIF)

Figure S2 **Proliferation rate and viability determined by trypan blue exclusion test.** PDLSCs, DPSCs and BMSCs display an increase in cell growth, time dependent. The Y-axis shows cell number, and X-axis shows culture time. Values obtained represent the average of three separate experiments ± SD.
(TIF)

Table S1 **Flow cytometry analysis of four different biological samples.**
(DOC)

Table S2 **Lists of high abundant assigned proteins.**
(DOC)
