# Supplementary figures and images for: Proteome of Human Stem Cells from Periodontal Ligament and Dental Pulp
Source: PLoS One. 2013 Aug 5;8(8):e71101. doi: 10.1371/journal.pone.0071101 (PMC3733711; doi:10.1371/journal.pone.0071101)

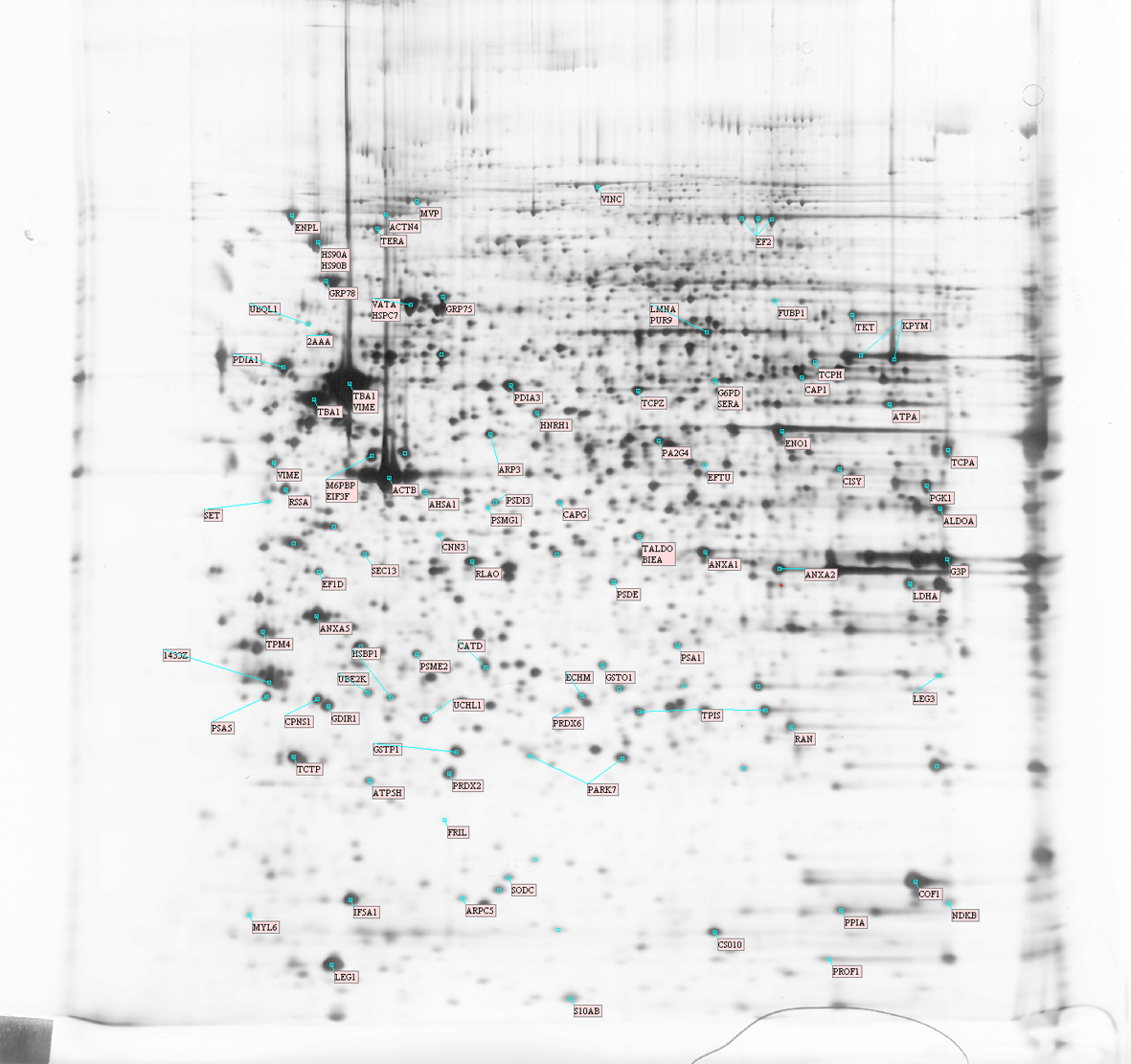

Supplement: Figure S1 — Localization on 2D map of high abundant proteins listed in table S2 and indicated by entry name. (TIF) [file pone.0071101.s001.tif]

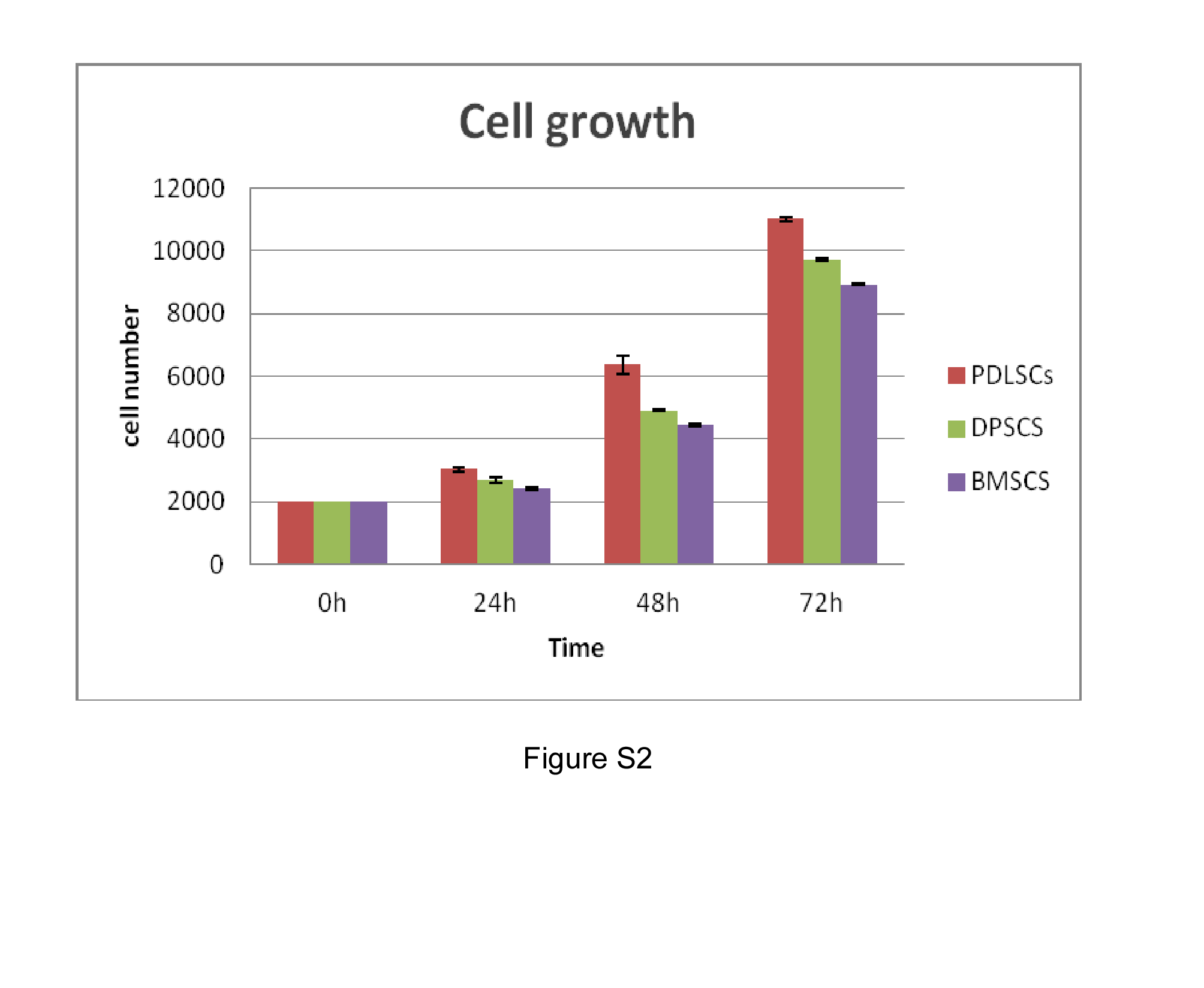

Supplement: Figure S2 — Proliferation rate and viability determined by trypan blue exclusion test. PDLSCs, DPSCs and BMSCs display an increase in cell growth, time dependent. The Y-axis shows cell number, and X-axis shows culture time. Values obtained represent the average of three separate experiments ± SD. (TIF) [file pone.0071101.s002.tif]
