# Supplementary material for: Proteome of Human Stem Cells from Periodontal Ligament and Dental Pulp
Source: PLoS One. 2013 Aug 5;8(8):e71101. doi: 10.1371/journal.pone.0071101 (PMC3733711; doi:10.1371/journal.pone.0071101)
Supplement: Table S1 — Flow cytometry analysis of four different biological samples. (DOC) [file pone.0071101.s003.doc]

| **Table S1 Flow cytometry phenotyping.** | | | | | | | |
| --- | --- | --- | --- | --- | --- | --- | --- |
| Antigens | | BMSCs | PDLSCs | DPSCs | BMSCs/PDLSCs | BMSCs/DPSCs | PDLSCs/DPSCs |
|  |  | MFI Ratio ± SD | | | p value | | |
| CD13 | **+** | 29,5±3,2 | 25,7±3,1 | 5,1±0,7 | 0,091 | ↓**0,004** | ↓**0,007** |
| CD14 | **-** | 1,2±0,1 | 1,2±0,1 | 1,3±0,2 | 0,302 | 0,084 | 0,037 |
| CD29 | **+** | 66,4±4,9 | 65,6±3,7 | 45,1±2,9 | 0,458 | 0,024 | ↓0,018 |
| CD34 | **-** | 1,1±0,1 | 1,2±0,1 | 1,1±0,1 | 0,289 | 0,211 | 0,301 |
| CD44 | **+** | 80,3±6,3 | 22,5±2,3 | 80,4±8,8 | ↓**0,004** | 0,494 | ↑**0,008** |
| CD45 | **-** | 1,2±0,1 | 1,2±0,2 | 1,2±0,3 | 0,483 | 0,426 | 0,435 |
| CD73 | **+** | 33,5±2,4 | 39,9±2,8 | 30,7±2,7 | ↑0,029 | 0,251 | ↓0,023 |
| CD90 | **+** | 83,8±9,4 | 162,9±16,8 | 66,1±5,1 | ↑**0,006** | 0,103 | ↓**0,005** |
| CD105 | **+** | 50,1±3,8 | 25,0±2,4 | 10,2±1,3 | ↓0,011 | ↓**0,003** | ↓0,012 |
| CD117 | **-/+** | 1,4±0,2 | 4,0±0,7 | 3,9±0,8 | ↑0,011 | ↑0,017 | 0,474 |
| CD133 | **-** | 1,2±0,1 | 2,1±0,3 | 1,4±0,2 | ↑0,013 | 0,095 | 0,078 |
| CD144 | **-** | 1,4±0,2 | 1,3±0,1 | 1,2±0,1 | 0,181 | 0,152 | 0,164 |
| CD146 | **+** | 10,1±0,9 | 10,4±1,2 | 5,4±0,5 | 0,394 | ↓0,018 | ↓0,017 |
| CD166 | **+** | 14,3±0,9 | 8,6±0,7 | 16,1±1,2 | ↓0,016 | 0,047 | ↑**0,003** |
| CD271 | **-** | 1,4±0,2 | 1,4±0,2 | 1,3±0,1 | 0,252 | 0,475 | 0,405 |
| OCT3/4 | **+** | 3,5±0,3 | 3,7±0,5 | 7,6±0,7 | 0,393 | ↑**0,009** | ↑0,015 |
| Sox-2 | **+** | 66,3±5,1 | 100,3±8,3 | 140,4±11,2 | ↑**0,010** | ↑**0,009** | ↑0,015 |
| SSEA-4 | **+** | 2,7±0,6 | 33,1±2,9 | 4,0±0,3 | ↑**0,003** | 0,031 | ↓**0,002** |
| MFI Ratio is the average of four different biological samples ± standard deviation; Cut-off Ratio positivity >1,5. Bold values represent the statistically significant variation of MFI Ratio (p≤ 0,01); up and down arrows represent surface expression increase or decrease trend respectively (p≤ 0,03); Cutoff Ratio positivity >2.0. | | | | | | | |
