# Supplementary material for: Proteome of Human Stem Cells from Periodontal Ligament and Dental Pulp
Source: PLoS One. 2013 Aug 5;8(8):e71101. doi: 10.1371/journal.pone.0071101 (PMC3733711; doi:10.1371/journal.pone.0071101)
Supplement: Table S2 — Lists of high abundant assigned proteins. (DOC) [file pone.0071101.s004.doc]

Table S2. Lists of high abundant assigned proteins

| **Abbreviation¥** | **Protein name** | **Accession  Number**  **©** | **Score §** | **% Sc ¤** | **# peptide matched** | **Exp. pI_MW** | **Theor. pI_MW** |
| --- | --- | --- | --- | --- | --- | --- | --- |
| 1433Z | 14-3-3 protein zeta/delta | P63104 | 128 | 54 | 21 | 4.69_26527 | 4.73_27899 |
| 2AAA | Serine/threonine-protein phosphatase 2A 65 kDa regulatory subunit A alpha isoform | P30153 | 165 | 37 | 21 | 5.33_63898 | 5.00_66065 |
| ACT4N | Alpha-actinin-4 | O43707 | 104 | 15 | 12 | 5.59_98590 | 5.27 _105245 |
| ACTB | Actin. cytoplasmic 1 | P60709 | 187 | 51 | 19 | 5.49_35891 | 5.29_42052 |
| AHSA1 | Activator of 90 kDa heat shock protein ATPase homolog 1 | O95433 | 167 | 57 | 18 | 5.60_33540 | 5.41_38421 |
| ALDOA | Fructose-bisphosphate aldolase A | P04075 | 113 | 42 | 14 | 8.94_33637 | 8.30_39851 |
| ANXA1 | Annexin A1 | P04083 | 210 | 69 | 26 | 6.21_35794 | 6.57_38918 |
| ANXA2 | Annexin A2 | P07355 | 320 | 79 | 34 | 7.76_36153 | 7.57_38808 |
| ANXA5 | Annexin A5 | P08758 | 216 | 70 | 23 | 5.14_35971 | 5.14_35971 |
| ARP3 | Actin-Related Protein 3 | P61158 | 129 | 39 | 17 | 5.76_47797 | 5.61_47497 |
| ARPC3 | Actin-related protein 2/3 complex subunit 3 | O15145 | 63 | 39 | 10 | 9.24_19266 | 8.78_20761 |
| ARPC5 | Actin-related protein 2/3 complex subunit 5 | O15511 | 70 | 53 | 9 | 5.83_18030 | 5.47_16367 |
| ATP5H | ATP synthase subunit d. mitochondrial | O75947 | 103 | 88 | 13 | 5.43_21818 | 5.21_18537 |
| ATPA | ATP synthase subunit alpha. mitochondrial | P25705 | 271 | 59 | 35 | 8.48_51870 | 9.16_59828 |
| BIEA | Biliverdin reductase A | P53004 | 76 | 30 | 10 | 6.34_35082 | 6.06_33692 |
| CAP1 | Adenylyl cyclase-associated protein 1 | Q01518 | 83 | 25 | 12 | 7.83_54818 | 8.27_52222 |
| CATD | Cathepsin D | P07339 | 74 | 30 | 11 | 5.82_27179 | 6.10_45037 |
| CH60 | 60 kDa heat shock protein. mitochondrial | P10809 | 211 | 71 | 48 | 5.51_59803 | 5.70_61187 |
| CLIC1 | Chloride intracellular channel protein 1 | O00299 | 182 | 57 | 14 | 5.41_30529 | 5.09_27248 |
| CNN2 | Calponin-2 | Q99439 | 102 | 46 | 11 | 6.94_36070 | 6.95_34074 |
| CNN3 | Calponin-3 | Q15417 | 77 | 30 | 12 | 5.60_34906 | 5.69_36562 |
| COF1 | Cofilin-1 | P23528 | 159 | 68 | 13 | 8.67_18525 | 8.22_18819 |
| CPNS1 | Calpain small subunit 1 | P04632 | 130 | 60 | 20 | 5.18_23595 | 5.05_28469 |
| EF1A1 | Elongation factor 1-alpha 1 | P68104 | 119 | 42 | 18 | 9.70_50541 | 9.10_50451 |
| EF1B | Elongation factor 1-beta | P24534 | 59 | 29 | 7 | 4.61_31180 | 4.50_24910 |
| EF1D | Elongation factor 1-delta | P29692 | 139 | 56 | 14 | 5.21_36159 | 4.90_31217 |
| EF2 | Elongation factor 2 | P13639 | 151 | 28 | 20 | 7.01_90374 | 6.41_96246 |
| EFTU | Elongation factor Tu. mitochondrial | P49411 | 263 | 64 | 34 | 6.41_40085 | 7.26_49852 |
| EIF3F | Eukaryotic translation initiation factor 3 subunit F | O00303 | 60 | 29 | 11 | 5.44_45739 | 5.24_37654 |
| ENOA | Alpha-enolase | P06733 | 331 | 71 | 35 | 7.08_48184 | 7.01_47481 |
| ENPL | Endoplasmin | P14625 | 152 | 37 | 32 | 4.76_92696 | 4.76_92696 |
| FRIL | Ferritin light chain | P02792 | 94 | 61 | 11 | 5.64_20556 | 5.51_20064 |
| FUBP1 | Far upstream element-binding protein 1 | Q96AE4 | 107 | 31 | 18 | 7.05_69340 | 7.18_67690 |
| G3P | Glyceraldehyde-3-phosphate dehydrogenase | P04406 | 67 | 26 | 11 | 8.96_36201 | 8.57_36201 |
| G6PD | Glucose-6-phosphate 1-dehydrogenase | P11413 | 245 | 56 | 26 | 6.34_54692 | 6.39_59675 |
| GDIR1 | Rho GDP-dissociation inhibitor 1 | P52565 | 119 | 51 | 13 | 5.34_22826 | 5.02_23250 |
| GRP75 | Stress-70 protein. mitochondrial | P38646 | 265 | 55 | 28 | 5.66_72335 | 5.85_73920 |
| GRP78 | 78 kDa glucose-regulated protein | P11021 | 256 | 56 | 38 | 5.35_74823 | 5.07_72408 |
| GSTO1 | Glutathione S-transferase omega-1 | P78417 | 101 | 40 | 12 | 6.23_27059 | 6.23_27833 |
| GSTP1 | Glutathione S-transferase P | P09211 | 169 | 60 | 15 | 5.85_19827 | 5.43_23569 |
| HNRH1 | Heterogeneous nuclear ribonucleoprotein H | P31943 | 242 | 69 | 32 | 6.07_50689 | 5.89_49884 |
| HS90B | Heat shock 90 B | P08238 | 106 | 37 | 24 | 5.33_85421 | 4.97_83554 |
| HSBP1 | Heat shock factor-binding protein 1 | O75506 | 60 | 39 | 8 | 5.98_22826 | 5.98_22926 |
| HSBP1 | Heat shock factor-binding protein 1 | O75506 | 91 | 49 | 11 | 5.50_23543 | 5.98_22826 |
| HSP7C | Heat shock cognate 71 kDa protein | P11142 | 189 | 47 | 23 | 5.56_70922 | 5.37_71082 |
| IF5A1 | Eukaryotic translation initiation factor 5A-1 | P63241 | 111 | 79 | 12 | 5.38_18030 | 5.08_17049 |
| KPYM | Pyruvate kinase isozymes M1/M2 | P14618 | 236 | 54 | 26 | 8.53_58470 | 7.96_58470 |
| LDHA | L-lactate dehydrogenase A chain | P00338 | 80 | 32 | 11 | 8.62_36108 | 8.44_36950 |
| LDHB | L-lactate dehydrogenase B chain | P07195 | 93 | 34 | 13 | 5.83_36167 | 5.71_36900 |
| LEG1 | Galectin-1 | P09382 | 170 | 82 | 12 | 5.34_15048 | 5.34_15048 |
| LEG3 | Galectin-3 | P17931 | 100 | 35 | 10 | 8.91_25662 | 8.56_26193 |
| LMNA | Lamin-A/C | P02545 | 295 | 58 | 43 | 6.23_63360 | 6.57_74380 |
| MOES | Moesin | P26038 | 84 | 23 | 14 | 6.23_65421 | 6.10_67892 |
| MVP | Major vault protein | Q14764 | 237 | 34 | 29 | 5.58_96972 | 5.34_99551 |
| MYL6 | Myosin light polypeptide 6 | P60660 | 68 | 43 | 7 | 4.56_17090 | 4.56_17090 |
| NDKA | Nucleoside diphosphate kinase A | P15531 | 126 | 60 | 13 | 5.91_19150 | 5.83_17309 |
| NDKB | Nucleoside diphosphate kinase B | P22392 | 114 | 71 | 14 | 9.01_17921 | 9.4_17401 |
| NPM | Nucleophosmin | P06748 | 67 | 41 | 14 | 4.84_35170 | 4.64_32726 |
| PA2G4 | Proliferation-associated protein 2G4 | Q9UQ80 | 128 | 52 | 25 | 6.41_48597 | 6.13_44101 |
| PARK7 | Protein DJ-1 | Q99497 | 69 | 49 | 9 | 6.27_20401 | 6.33_20050 |
| PARK7 | Protein DJ-1 | Q99497 | 88 | 52 | 10 | 5.90_20248 | 6.33_20050 |
| PDIA1 | Protein disulfide-isomerase | P07237 | 407 | 70 | 45 | 4.79_57601 | 4.76_57480 |
| PDIA3 | Protein disulfide-isomerase A3 | P30101 | 315 | 56 | 33 | 5.85_54629 | 5.98_57196 |
| PGK1 | Phosphoglycerate kinase 1 | P00558 | 268 | 76 | 38 | 8.81_35763 | 8.30_44935 |
| PLIN3 | Perilipin 3 | O60664 | 161 | 60 | 22 | 5.44_45739 | 5.30_47189 |
| PPIA | Peptidyl-prolyl cis-trans isomerase A | P62937 | 187 | 81 | 20 | 7.86_17666 | 7.68_18229 |
| PPIB | Peptidyl-prolyl cis-trans isomerase B | P23284 | 163 | 64 | 23 | 9.24_19266 | 9.42_23785 |
| PRDX1 | Peroxiredoxin 1 | Q06830 | 170 | 78 | 20 | 8.86_21129 | 8.27_22324 |
| PRDX2 | Peroxiredoxin 2 | P32119 | 108 | 35 | 9 | 5.66_22049 | 5.66_22049 |
| PRDX6 | Peroxiredoxin 6 | P30041 | 121 | 70 | 16 | 6.07_26849 | 6.00_25133 |
| PROF1 | Profilin-1 | P07737 | 165 | 87 | 14 | 7.69_15698 | 8.44_15216 |
| PSA1 | Proteasome subunit alpha type-1 | P25786 | 119 | 66 | 15 | 6.15_29822 | 6.15_29822 |
| PSA5 | Proteasome subunit alpha type-5 | P28066 | 60 | 59 | 10 | 4.71_24016 | 4.74_26565 |
| PSDE | 26S proteasome non-ATPase regulatory subunit 14 | O00487 | 128 | 41 | 21 | 6.22_36106 | 6.06_34726 |
| PSME1 | Proteasome activator complex subunit 1 | Q06323 | 151 | 75 | 22 | 5.81_27360 | 5.78_28876 |
| PSME2 | Proteasome activator complex subunit 2 | Q9UL46 | 146 | 49 | 16 | 5.57_29106 | 5.44_27515 |
| PSMG1 | Proteasome assembly chaperone 1 | O95456 | 61 | 28 | 6 | 5.77_33595 | 6.88_33631 |
| PUR9 | Bifunctional purine biosynthesis protein PURH | P31939 | 76 | 37 | 15 | 6.23_63360 | 6.27_65089 |
| RAN | GTP-binding nuclear protein Ran | P62826 | 114 | 64 | 16 | 7.17_23767 | 7.01_24579 |
| RLA0 | 60S acidic ribosomal protein P0 | P05388 | 138 | 52 | 14 | 5.92_36191 | 5.71_34423 |
| RSSA | 40S ribosomal protein SA | P08865 | 170 | 41 | 13 | 4.79_32947 | 4.79_32947 |
| S10AB | Protein S100-A11 | P31949 | 59 | 57 | 7 | 6.07_11500 | 6.56_11847 |
| SEC13 | Protein SEC13 homolog | P55735 | 146 | 38 | 14 | 5.43_35637 | 5.22_36031 |
| SERA | D-3-phosphoglycerate dehydrogenase | O43175 | 81 | 27 | 12 | 6.34_54692 | 6.29_57367 |
| SET | Protein SET | Q01105 | 100 | 26 | 8 | 4.39_33891 | 4.23_33469 |
| SODC | Superoxide dismutase [Cu-Zn] | P00441 | 61 | 39 | 5 | 5.80_18652 | 5.70_16154 |
| STMN1 | Stathmin | P16949 | 126 | 69 | 16 | 5.78_18290 | 5.76_17292 |
| TALDO | Transaldolase | P37837 | 150 | 41 | 18 | 6.06_37692 | 6.36_37688 |
| TALDO | Transaldolase | P37837 | 199 | 47 | 25 | 6.03_35907 | 6.36_37688 |
| TBA1B | Tubulin alpha-1B chain | P68363 | 105 | 48 | 15 | 5.39_54128 | 4.94_50804 |
| TBB5 | Tubulin beta chain | P07437 | 227 | 62 | 32 | 5.11_52956 | 4.78_50095 |
| TCPA | T-complex protein 1 subunit alpha | P17987 | 77 | 16 | 8 | 6.05_60652 | 5.80_60819 |
| TCPE | T-complex protein 1 subunit epsilon | P48643 | 168 | 49 | 33 | 5.65_59719 | 5.45_60089 |
| TCPH | T-complex protein 1 subunit eta | Q99832 | 131 | 34 | 21 | 7.87_56680 | 7.55_59842 |
| TCPZ | T-complex protein 1 subunit zeta | P40227 | 140 | 52 | 24 | 6.33_53693 | 6.23_58444 |
| TCTP | Translationally-controlled tumor protein | P13693 | 122 | 54 | 13 | 4.84_19697 | 4.84_19697 |
| TERA | Transitional endoplasmic reticulum ATPase | P55072 | 350 | 54 | 43 | 5.47_89487 | 5.14_89950 |
| TKT | Transketolase | P29401 | 170 | 49 | 28 | 8.01_67224 | 7.58_68519 |
| TPIS | Triosephosphate isomerase | P60174 | 199 | 81 | 16 | 6.85_26585 | 6.45_26938 |
| TPIS | Triosephosphate isomerase | P60174 | 87 | 57 | 12 | 6.07_26849 | 6.45_26938 |
| TPM4 | Tropomyosin alpha-4 chain | P67936 | 180 | 48 | 18 | 4.70_33294 | 4.67_28619 |
| UBE2K | Ubiquitin-conjugating enzyme E2 | P61086 | 78 | 30 | 6 | 5.50_23794 | 5.33_22507 |
| UBE2N | Ubiquitin-conjugating enzyme E2 N | P61088 | 121 | 55 | 12 | 6.01_17090 | 6.13_17184 |
| UBQL1 | Ubiquilin-1 | Q9UMX0 | 77 | 25 | 11 | 5.06_65632 | 5.02_62479 |
| UCHL1 | Ubiquitin carboxyl-terminal hydrolase isozyme L1 | P09936 | 161 | 70 | 15 | 5.59_25723 | 5.33_25151 |
| VATA | V-type proton ATPase catalytic subunit A | P38606 | 71 | 22 | 9 | 5.56_70922 | 5.35_68360 |
| VIME | Vimentin | P08670 | 415 | 70 | 52 | 4.73_43823 | 5.06_53676 |
| VIME | Vimentin | P08670 | 295 | 66 | 41 | 5.39_54128 | 5.06_53076 |
| VINC | Vinculin | P18206 | 227 | 30 | 32 | 6.22_101017 | 5.32_124292 |
| WDR1 | WD repeat-containing protein 1 | O75083 | 124 | 40 | 19 | 6.21_65263 | 6.17_66836 |

**¥** **Abbreviation** It Is omitted the _Human.

**§ Score** It is -log_10_(P), where P is probability that the observed mach is a random event, it is based on Swiss Prot database using the MASCOT searching program

¤ **% Sc** Sequence coverage means the ratio of portion sequence covered by matched peptide to the full length of the protein sequence.

**© Accession Number** in integrated UniProtKB/Swiss-Prot.
